# Investigating effects of acute severe hypoxia and p38 MAPK inhibition on boldness and anxiety-like behavior in zebrafish larvae

**DOI:** 10.3389/fnbeh.2026.1717856

**Published:** 2026-03-18

**Authors:** Karem Vazquez-Roman, Warren Burggren

**Affiliations:** Developmental Integrative Biology Research Group, Department of Biological Sciences, University of North Texas, Denton, TX, United States

**Keywords:** anxiety-like behavior, boldness, cardiac injury, exploratory behavior, hypoxia, p38 MAPK inhibition, zebrafish

## Abstract

Severe tissue hypoxia, resulting from cardiac dysfunction for example, is increasingly recognized as a trigger for neurobehavioral change. However, the impact of tissue hypoxia on anxiety-like behavior and disinhibition during development remains poorly understood. Zebrafish larvae provide an effective vertebrate model to evaluate how hypoxia influences behavior in early life. We assigned larvae to three groups: (1) normoxic control, (2) acute severe hypoxic exposure (HE) using cardiac dysfunction as evidence of exposure severity, or (3) HE plus 12 h exposure to 0.3 μmol AZ3 (p38 MAPK inhibitor). At one- and 2-weeks post-exposure we quantified survival, morphometrics, and behavioral measurements (emergence latency, thigmotaxis, zone occupancy, novel object approach, and velocity). Survival differed significantly among all groups (*P* < 0.001). Untreated HE reduced occupancy of the novel object zone 21% relative to controls at 2 weeks (*P* = 0.04), indicating diminished neophilia (attraction to novel objects). p38 MAPK inhibition increased body mass (*P* < 0.001 vs. controls) and prevented the length restriction seen in HE, but also increased by 17% the time spent in thigmotaxis zone compared to HE larvae alone (*P* = 0.04), consistent with increased anxiety-like behavior. Neither emergence latency nor velocity differed between groups, but both variables changed in the three groups after 1 week decreasing and increasing respectively. Novelty approach distance was unaffected by treatment. Thus, acute early-life hypoxic exposure in zebrafish—severe enough to induce cardiac injury among other forms of likely tissue damage—results in statistically significant but modest decreases in exploratory behavior. P38 MAPK inhibition stimulates growth compared to controls, and also decreases exploratory behavior compared to hypoxic exposure alone and promotes small anxiety-like responses without altering general locomotion. The results highlight the importance of integrating behavioral endpoints into hypoxia exposure research and suggest that targeting p38 MAPK may carry neurobehavioral trade-offs.

## 1 Introduction

Both the vertebrate heart and the brain are very high-energy demanding organs, relying on high oxygen requirements to produce sufficient ATP to sustain metabolic processes (Mir et al., 2025; Özugur et al., 2020; Ventura-Clapier et al., 2011; Watts et al., 2018). The tight coupling between neuronal activity and substrate delivery becomes disrupted when cerebral blood flow decreases (Kanat et al., 2003). Such disruption can arise for a variety of reasons, often a result of cardiac dysfunction. Systemic exposure to acute severe hypoxia in zebrafish larvae, resembling myocardial infarction, causes oxidative damage to multiple organs, including brain (Napolitano et al., 2018; Sawahata et al., 2021; Silva et al., 2016) and heart (Burggren et al., 2024; Zou et al., 2019). The cardiovascular parameters that contribute to neurological consequences extend far beyond simple “pump failure,” after MI, encompassing blood pressure dynamics, inflammatory signaling, barrier function, and cerebrovascular regulation—all of which must be considered when evaluating and treating humans with cardiac disease at risk for neurological complications.

We focused on larval zebrafish to investigate whether acute hypoxic injury during a critical developmental window—when cardiac and neural systems are actively maturing—establishes lasting behavioral phenotypes. Early-life exposure paradigms are particularly relevant for understanding developmental origins of adult disease and behavioral disorders.

One of the poorly explored impacts of the complex relationship between brain function and cardiac perfusion is subtle changes in behavior. Myocardial infarction has long been recognized as a condition inducing behavioral changes, for example inducing psychological stress and behavioral impairment in humans (Murphy et al., 2020; Murphy et al., 2015) and reducing exploratory behavior in experimental models (Schoemaker and Smitst, 1994). Presumably, the main effector is the severe tissue hypoxia resulting from cardiac dysfunction. However, little is known from the clinical perspective specifically regarding “boldness” in humans with cardiovascular diseases leading to tissue hypoxia. Anxiety-like behavior was evaluated in rats after myocardial infarction, with epigenetic disruption of the hippocampus being one of the mechanisms involved in increasing anxiety (Zhou et al., 2020).

Anxiety-like behavior and boldness represent two closely related yet distinct dimensions of animal personality and affective function (Khursigara et al., 2024). Anxiety is characterized by heightened caution, avoidance, and increased stress responses to perceived threats. Boldness reflects an individual’s propensity to take risks and explore novel environments. Boldness (and its counterpart, shyness) is included in a framework of 16 personality factors (Cattell and Mead, 2008). Boldness behavior is also associated with exploration in animal behavior (Dean et al., 2020; Hamilton et al., 2021; Ólafsdóttir and Magellan, 2016; Wisenden et al., 2011). We employed the novel object approach test, validated for measuring neophilia in 14–21 dpf zebrafish larvae, with maximal responses observed at 14 dpf. Neophilia—attraction to novel objects—serves as an established proxy for boldness in fish behavior, reflecting risk-taking propensity and exploratory drive (Dahlbom et al., 2011; Dean et al., 2020). Thigmotaxis (wall-hugging behavior) is validated as an anxiety-like measure in zebrafish larvae from 7 dpf onward (Richendrfer et al., 2012; Schnörr et al., 2012). These behavioral constructs are evolutionarily conserved across vertebrates, facilitating translation to mammalian systems.

Our main aim was to explore the link between acute systemic tissue hypoxia and subsequent chronic behavioral effects in the zebrafish larvae. We used cardiac arrest and associated myocardial damage as a proxy for hypoxia-induced systemic damage—essentially, modeling effects of human myocardial infarction, rather than chronic cardiac disease. We tested the hypothesis in the zebrafish larval model that acute systemic tissue hypoxia will cause subsequent chronic changes in behavior as the larvae continue to grow and develop. We also tested the effects on hypoxia-related behavioral alterations of p38 mitogen-activated protein kinase (p38 MAPK). P38 MAPK is involved in multiple body systems, responding mainly to stress, and inflammation (Wang et al., 2024; Zhang et al., 2007). P38 MAPK plays essential roles in cardiovascular, nervous, immune, hepatic, gastrointestinal, and reproductive systems (Han et al., 2020). This protein kinase has been proposed as a therapeutical target to induce cardiac regeneration, being a stress-responsive kinase that regulates cardiac inflammation, cell death, hypertrophy, and contractility (Bassi et al., 2008). However, there is still no supporting clinical evidence as a treatment for any inflammatory diseases (Wang et al., 2024). Furthermore, there are no studies addressing myocardial recovery plus behavioral evaluation (as part of potential side effects) involving protein kinases (Asih et al., 2020; Corrêa and Eales, 2012). Similar to its role in cardiac tissue, p38α is also the most extensively studied p38 isoform in the brain (Asih et al., 2020; Corrêa and Eales, 2012). Based on our previous experiments, where cardiac function increased in larvae experiencing myocardial hypoxic injury after p38 MAPK inhibition (Vazquez Roman, 2024) (article in preparation), we have analyzed this compound’s behavioral effects on larvae following systemic tissue hypoxia severe enough to cause cardiac damage and arrest. We tested the hypothesis that p38 inhibition will modify the behavioral changes (anxiety-like and boldness-like behaviors) in zebrafish larvae produced by hypoxic injury.

The zebrafish as a model offers a tractable *in vivo* system to jointly assess the effects of severe hypoxia on behavior. By quantifying thigmotaxis, emergence latency, swimming velocity, exploration, and novel-object approach at 1- and 2-weeks post-hypoxic exposure, our study sought to correlate acute severe tissue hypoxia, cardiac damage, pharmacologic recovery, and behavioral effects into an integrated framework that can inform mechanisms, side-effect profiles, and potential prognostic markers.

## 2 Material and method

### 2.1 Zebrafish sources, husbandry and breeding

Adult wild-type (AB strain) zebrafish were obtained from a commercial supplier in Denton, Texas. All adult fish were maintained under the same controlled conditions: Aquarium water containing 60 mg of Instant Ocean Aquarium Sea Salt per 1 liter of Deionized (DI) Water, maintained at 28 ± 0.5°C, with 14:10 h light:dark photoperiod, fed twice a day (∼3% body weight) with Tetramin Tropical Flakes and once a day with frozen brine shrimp (*Artemia* sp.) on nauplii stage. Water quality parameters were assessed daily (pH 7–7.6, ammonia, nitrates and nitrites at ∼0 ppm). Healthy adult zebrafish (4–6 months old) were used as breeding stock in the Aquatic Facilities at the University of North Texas. Females and males (∼100 fish per tank) were kept together in large glass tanks (∼90 L).

Zebrafish typically breed when the laboratory lights turn on (08:00). During the night before the planned breeding, small breeding tanks with artificial plastic plants as enrichment were placed attached to the inside glass wall of the larger tanks. At breeding, adult fish came and swam above the breeding tanks. Hence, as a result of multiple breeding pairs, large numbers of fertilized eggs fell to the bottom of the breeding tanks and were collected and washed with aquarium water.

Viable embryos were separated from infertile eggs and reared in E3 media. All surviving embryos hatched at ∼4 dpf. The larvae were fed twice daily with Otohime*™* A1 Starter larvae food, alternated with A2 at 14 dpf (PTAqua, Dublin, Ireland), sprinkled on the medium surface. Larvae were always held in approximately the same density (∼50 larvae/L) to avoid differences in growth that can emerge from crowding. They were reared in static tanks with water changed twice a day.

All experiments were conducted under University of North Texas Institutional Animal Care and Use Committee permit # 18005.

### 2.2 Hypoxia and p38 MAPK inhibitor exposures

Zebrafish larvae were divided into three groups at 7 dpf. The first group—designated the controls—was held in normoxic aquarium water and never exposed to hypoxia or p38 MAPK inhibitor treatment. Boldness in zebrafish can be dictated by both the strain of zebrafish and the test context (Mustafa et al., 2019). Consequently, a control group was used in every replicate to reduce the effect caused by strain/age of parents, or other extrinsic factors influencing variation as a confounding result.

**FIGURE 1:**
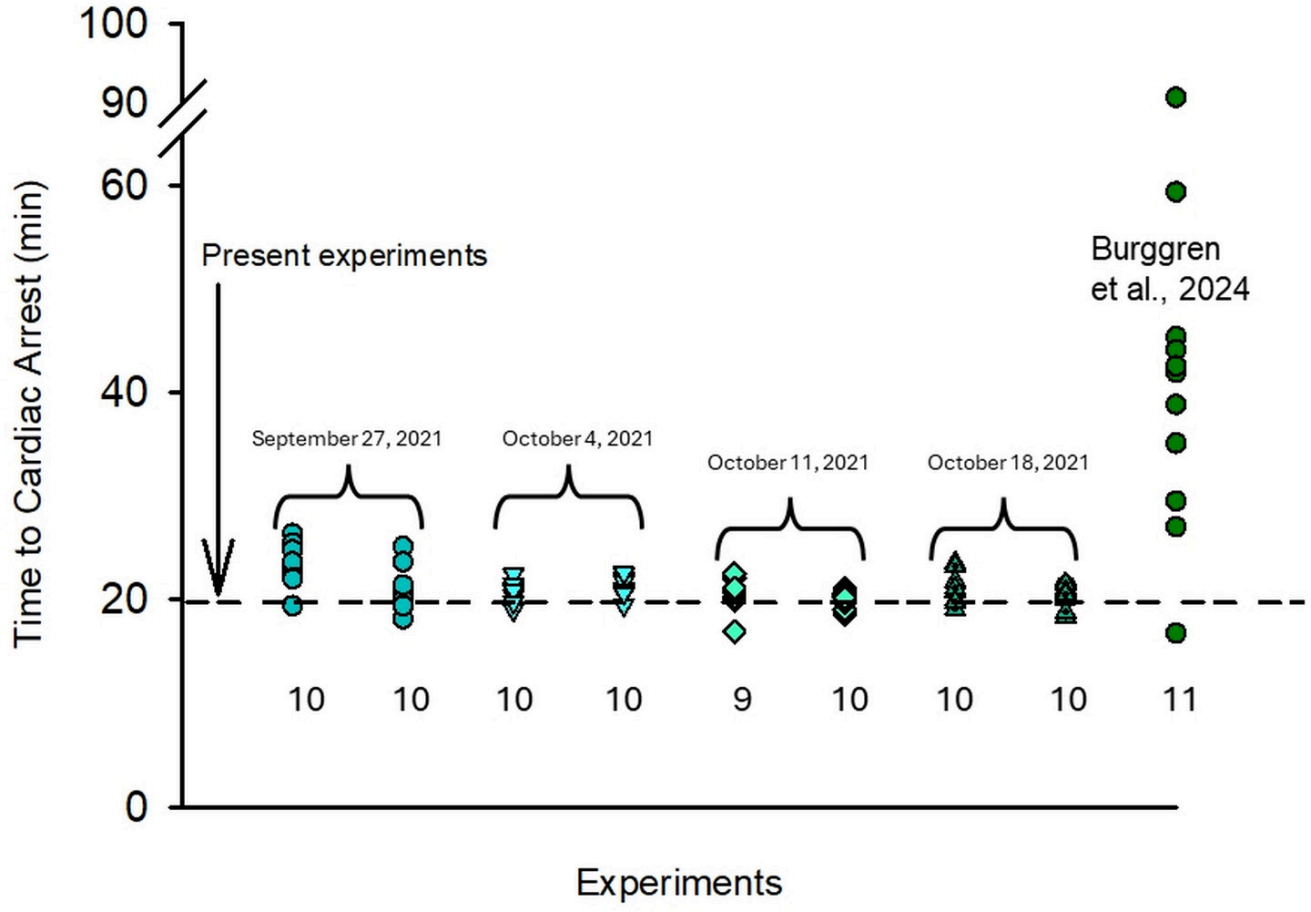
Comparison time taken by zebrafish larvae to reach cardiac arrest during acute severe (1 KPa O_2_) hypoxic exposure. Cardiac arrest, readily observed in larval zebrafish through the translucent body wall, was used as a proxy for systemic hypoxic damage. Individual larval data are plotted. N values are indicated below each experiment. Measurements on each provided date are from larvae derived from a separate clutch of zebrafish eggs.

The second group was exposed to acute severe ambient hypoxia (∼1 kPa) for a period of ∼20 min. This was a time judged to be sufficient to induce at least myocardial hypoxic injury. Both our preliminary experiments and previously published research showed that zebrafish larvae experience cardiac arrest after ∼20 min in severe hypoxic exposure (Figure 1; Burggren et al., 2024). This acute severe hypoxic exposure results not only in cardiac arrest, but also causes cardiomyocyte apoptosis, determined by observation of colocalization of molecular markers Cardiac Troponin T and Cleaved Caspase-3 (Burggren et al., 2024). Very likely additional tissues, including those of the brain and other neural tissue, are similarly damaged by this level of systemic hypoxia (Braga et al., 2013; Zeng et al., 2022). In the current experiment, pooled zebrafish larvae from the same clutch (∼100 larvae enclosed in a submerged glass chamber) were exposed to ∼1 KPa for ∼20 min. Following exposure, the pooled larvae were returned to normoxic water. Larvae under these acute severe hypoxic conditions were presumed to experience cardiovascular damage (if not cardiac arrest and possible subsequent death) due to the identical treatment as the larval zebrafish in the study of Burggren et al. (2024). This second group received no p38 MAPK inhibitor treatment.

The third larval zebrafish group was exposed to acute severe hypoxia causing presumed severe hypoxic injury (HE) as indicated for the second group described above. Cardiac arrest and heart damage was used as a proxy for systemic tissue damage following acute severe hypoxic exposure. However, this population was also exposed to 0.3 μmol of the protein kinase p38 MAPK inhibitor (AstraZeneca compound AZ3, 503.6 g/mol), in their surrounding medium (E3 or aquarium water, depending upon age) during the first 12 h following acute severe hypoxic exposure. This dose of p38 MAPK inhibitor and length of exposure was chosen because other experiments revealed that this dose induced the highest recovery of cardiac function in HE larvae (Vazquez-Roman *et al.*, in preparation). After 12 h of exposure to p38 MAPK inhibitor in E3 medium, treated larvae were placed in aquarium water until the day of testing.

### 2.3 Rearing conditions after hypoxic exposure and before behavioral trials

Immediately following acute severe hypoxic exposure and the resulting cardiac arrest and presumed myocardial hypoxic injury, all larvae were returned to normoxic E3 media, in which they were subsequently reared until day 8 dpf, at which point in development they were transferred to aquarium water. All larvae were fed with A1 starter larvae food twice daily; this feeding regime was alternated with A2 (Otohime*™* PTAqua, Dublin, Ireland) after they reached 14 dpf. Larvae were not fed during the days of behavioral testing.

### 2.4 Survival assessment

Survival was assessed daily over a 14 day period, beginning from immediately before hypoxic exposure at Day 7, until the last day of behavioral trials on Day 21. These days of hypoxic exposure, expressed as dHE, are equivalent to days of development (days post fertilization, dpf) as follows: 1 dHE = 7 dpf or 1 week following hypoxic exposure, and 15 dHE = 21 dpf or 2 weeks following hypoxic exposure.

### 2.5 Morphometric variables

Immediately after conclusion of the behavioral trials, larvae were fixed in Z-Fix (Electron Microscopy Sciences, Hatfield PA, United States). Body mass was determined after fixation, following a standardized procedure (Vazquez Roman and Burggren, 2022). Body mass measurements were conducted using an analytical balance (MettlerToledo XA105 Dual Range^§^). Total body length was assessed after larval whose body mass was recorded. Photographs for determining body length were obtained with a microscope connected to a high-resolution camera and analyzed in ImageJ.

After assessment of body mass and total body length, evaluation of larvae growth was conducted using the formula for Fulton’s Condition Factor:

*K* = 100 (W/L^3^)

where “W” represents body mass (weight), and “L” the total length (Froese, 2006).

### 2.6 Behavioral testing: anxiety-like and boldness-like behavior

Anxiety-like and boldness-like behavior are common measurements in fish behavior and may be employed in behavioral neuroscience (Cueto-Escobedo et al., 2022; Dean et al., 2020; Hagen et al., 2024). Zebrafish larvae were assessed at two different time points to evaluate anxiety-like behavior and boldness-like behavior. Behavioral testing was conducted at 14–15 dpf (1 week after HE), and 21–22 dpf (2 weeks after HE) to assess differences between exposed larvae and control larvae at these two different developmental points. Different larvae were used for each group of experiments—i.e., there were no repeated measurements.

**FIGURE 2:**
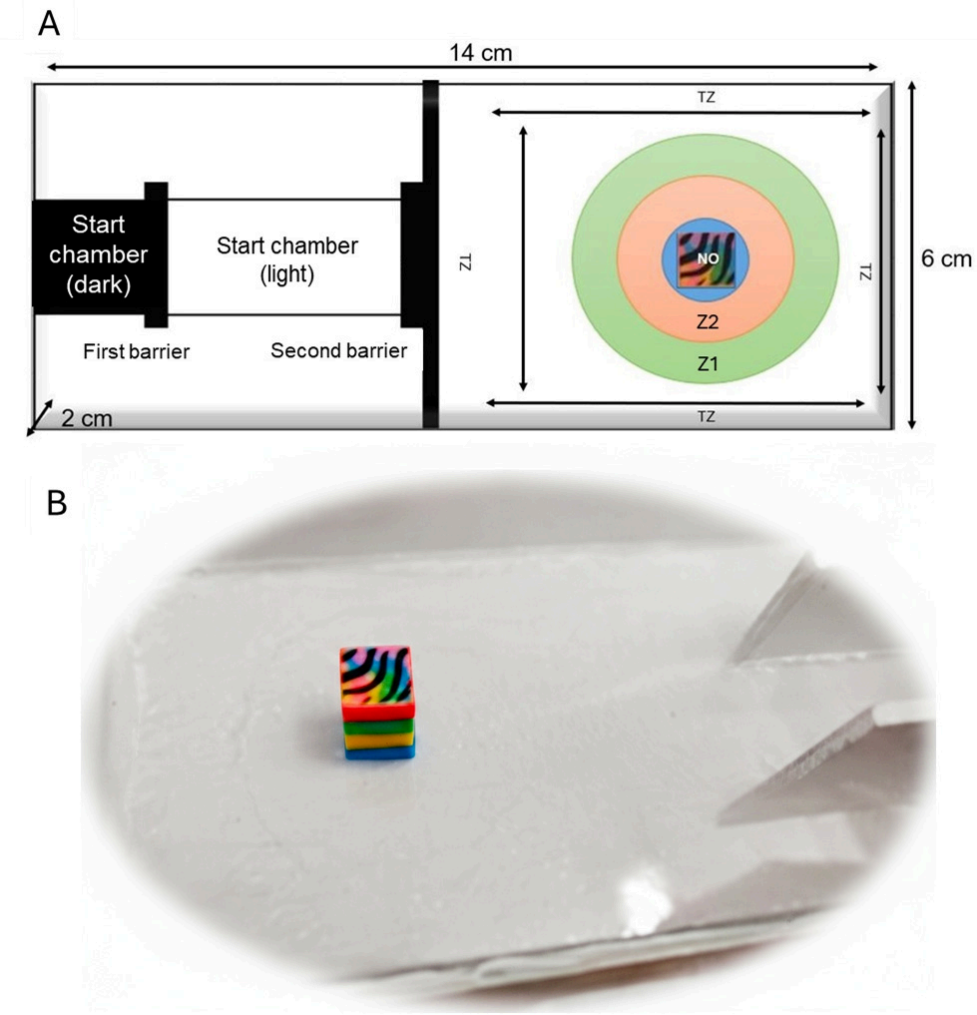
Testing arena and novel object. **(A)** Testing arena for novel object approach. NO, novel object; TZ, thigmotaxis zone; Z1, zone 1; Z2, zone 2. **(B)** Novel object seen from a lateral view.

The behavioral arena was identical to that used from Dunton et al. (2025), with multiple chambers leading into the actual test arena. This apparatus consisted of a dark start chamber and a light start chamber—both attached to an acrylic arena (14 cm long × 6 cm wide × 2 cm depth) (Figure 2A). The overall apparatus was filled to a depth of ∼1.8 cm with water maintained at 28 ± 0.5°C. A high-speed video camera (Logitech C920 × HD Pro Webcam, Full HD) was located ∼30 cm above the arena to record videos of larval behavior. Opaque cardboard was placed strategically covering the surroundings of the recording site, to avoid any visual influence (including by the experimenter) on the larvae.

For testing anxiety-like and boldness-like behaviors, an individual larva was placed into the dark start chamber. After a 3 min acclimation period to the dark start chamber, the opaque barrier separating was carefully removed without disturbing the larva. This enabled the larva potentially to swim into the attached light chamber. During a subsequent 3 min period, the individual larva was allowed to acclimate in the light chamber, and then the second opaque barrier separating light chamber and test arena was carefully removed to allow the novel object approach test to begin.

The novel object approach test was used to measure “neophilia,” the willingness to face new experiences and new environments. In fish this is a well-established proxy for boldness (Dahlbom et al., 2011; Dean et al., 2020; Hagen et al., 2024), with validation extending to larval stages at 14–21 dpf (Gjinaj et al., 2025). Boldness assessment was determined by the quantification of neophilia by determining the time spent near a novel object within a test arena. Thus, a longer time spent near the novel object signaled a greater degree of neophilia. The novel object comprised a tridimensional multicolored object formed from a 1 cm x 1 cm assembly of blue, yellow, green, and red LEGO “dots” ^®^. An array of colors was used to avoid bias related to color preference in zebrafish (Figure 2B). Multicolored LEGO^®^ figurines are useful in examining fish behavior, as they serve as effective, easily acquired and standardized novel objects to test how fish respond to unfamiliar items in their environment (Hamilton et al., 2016, 2021).

Video recordings (VirtualDub Software) started when the second door was removed. Videos were recorded for a 10 min period. The start chamber was immediately blocked off after the individual larva emerged (Figure 2A). Larvae that did not emerge during the 10 min-trial were discarded from the behavioral analyses.

For all behavioral tests only “naïve” larvae were used—i.e., no larvae were experimented upon twice.

### 2.7 Behavioral video analyses

EthoVision XT Noldus software with the DanoVision extension was used to analyze video recordings of zebrafish larval behavior. Concentric circles were added in EthoVision to virtually divide the videos of the arena into four different zones based on the position of the novel object (Figure 2A). The first zone added was zone 3 (surrounding the novel object), and the subsequent zones were created with an enlargement factor of 2 and 3 for zones 2, and 1, respectively. The exploration behavior in a novel tank was also used to measure boldness-exploration (Mazué et al., 2015).

Length of time (s, seconds) spent in the thigmotaxis zone (arena walls) was quantified to evaluate anxiety-like behavior. Time to emerge from the chamber, time spent in the novel object zone, and average distance to the object were compared as a proxy for boldness in zebrafish larvae. Average swimming velocity, related to locomotion, was also quantified.

### 2.8 Statistics

Survival analyses were conducted using a Kaplan-Meier Survival Analysis: LogRank test. General linear models (GLM) were generated for assessing time spent in each zone (thigmotaxis zone, zones 1–3, from outer to inner zones, respectively), and average distance to the object. Time of emergence was used as a covariate with time spent on each zone, and distance from the novel object. Normality of each model was assessed with the Shapiro Wilk test. Data from models that were not normally distributed were rank-transformed. Two-way ANOVAs were generated for this model. A Tukey *post-hoc* test was performed to find differences among groups and across time after acute severe hypoxic exposure. Results were reported as estimated marginal means (EMMs) ± S.E.M. when covariant was significant in the two-way ANOVA. If covariant was not significant for the particular dependent variable, results were plotted as means ± S. E. M. Survival analyses were performed with SigmaPlot software (Systat Software, Inc.^®^, San Jose, CA). R-Studio was used to perform two-way ANOVAs for Fulton’s Condition Factor, and time to emerge from start chamber and velocity, and Two-Way ANOVAs with emergence time as a covariate (time spent in each zone, and average distance to the object).

**FIGURE 3:**
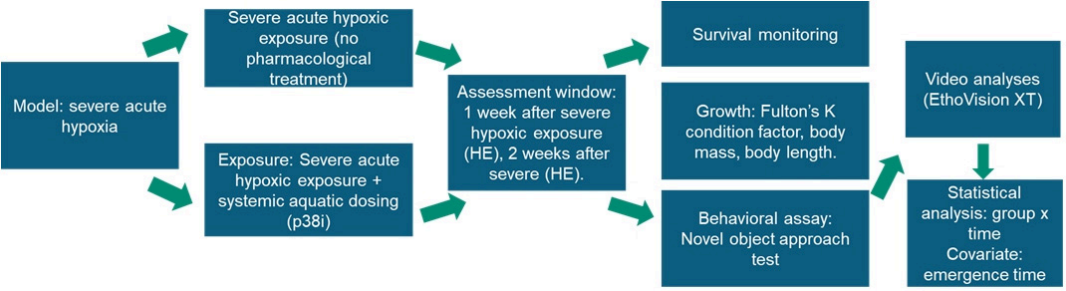
Flowchart overview of the experiments.

A significance level of *p* < 0.05 was adopted for all tests. The logic flow of the experiments is shown in Figure 3.

## 3 Results

### 3.1 Survival rate

**FIGURE 4:**
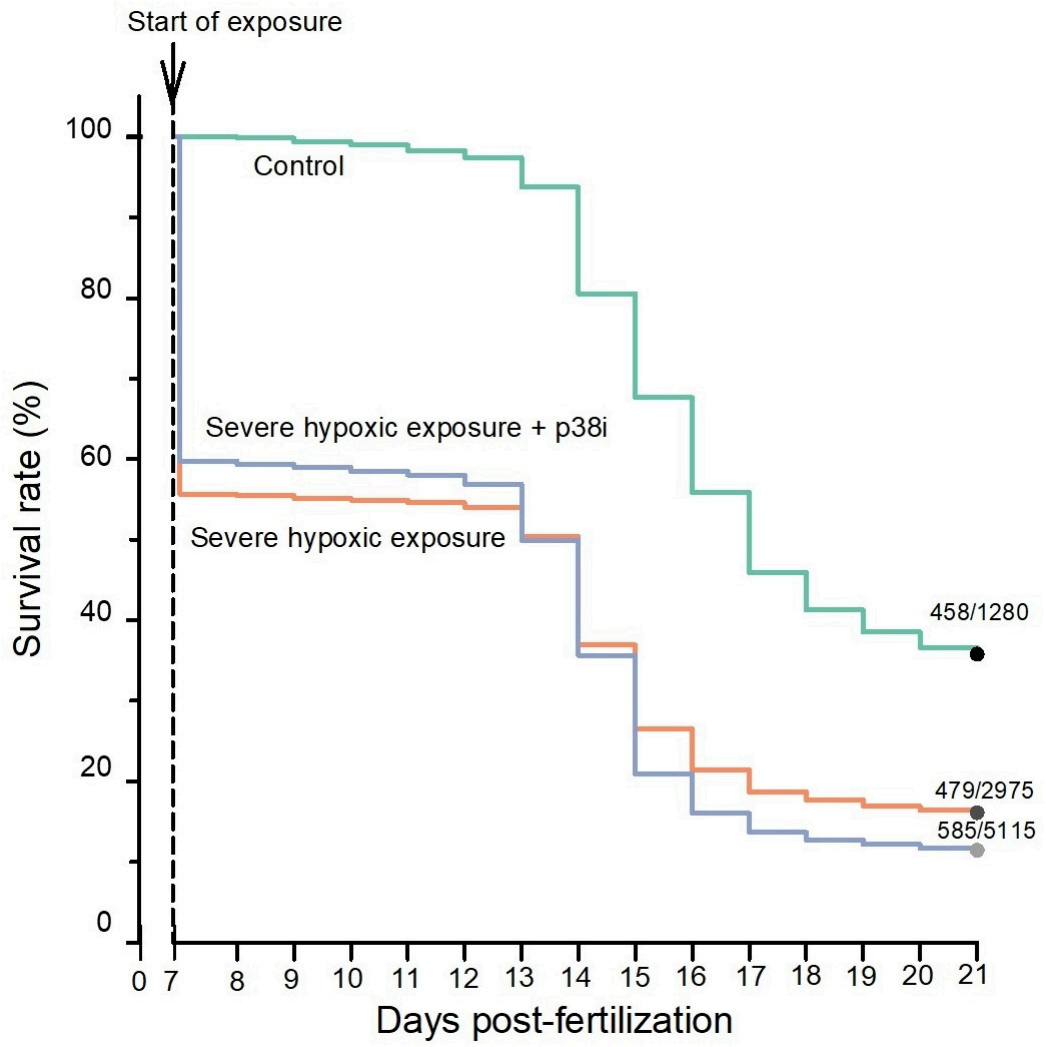
Survival assessment following acute severe systemic hypoxia—evident from myocardial hypoxic injury—and the effect of p38 MAPK inhibition in zebrafish larvae. All groups are significantly different from each other at one or more points following systemic hypoxic exposure (*P* < 0.001). See text for additional explanation.

Survival rate of the control population, the population experiencing tissue hypoxic damage (as evident from myocardial damage), and the hypoxic exposure + p38 MAPK inhibition population were determined based on the time after acute severe hypoxic exposure (Figure 4). Hypoxic exposure produced survival rates of 56% in the HE group and 60% in the HE + p38 MAPK inhibition group the day after hypoxic exposure. Two weeks after acute severe hypoxic exposure, a higher survival rate occurred in control groups (38%), followed by the HE group (16%), and lastly, the HE + p38 MAPK inhibition group (11%). All Pairwise Multiple Comparison Procedures revealed significant (*P* = < 0.001) differences among all group interactions: controls vs. HE, controls vs. HE + p38 MAPK inhibition, and HE vs. HE + p38 MAPK inhibition.

### 3.2 Effects of acute severe hypoxic exposure on growth

**FIGURE 5:**
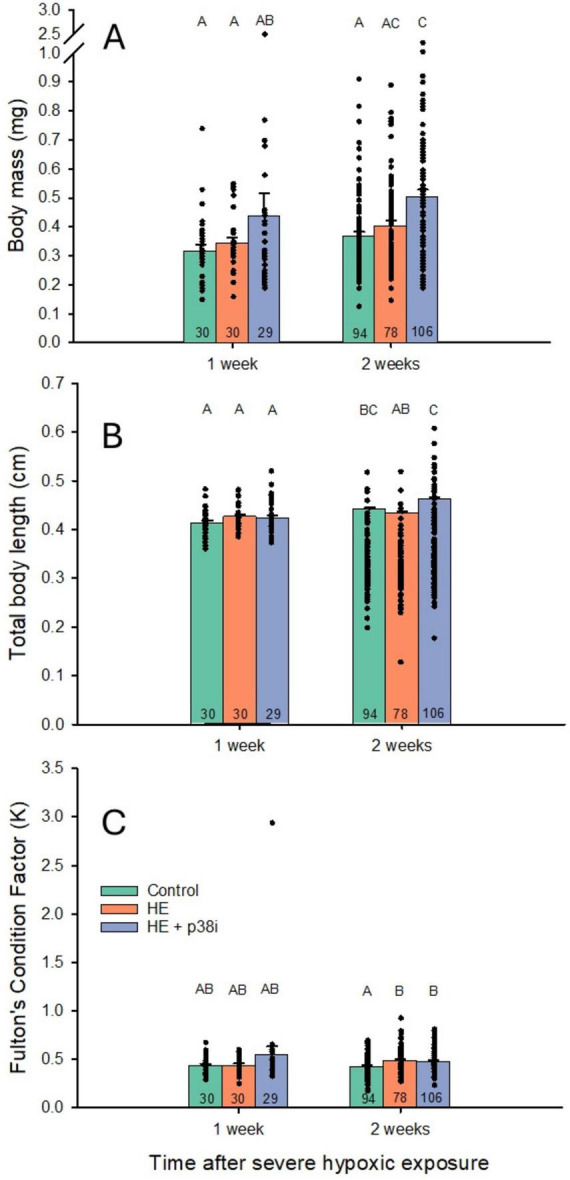
Effects of acute severe systemic hypoxic exposure on subsequent growth in zebrafish larvae. N values are at the bottom of each bar. Legends for the three graphs are indicated in **(C)**. **(A)** Larvae experiencing severe hypoxic exposure (HE) + p38 MAPK inhibition were significantly heavier than the control group at 2 weeks after severe hypoxic exposure (*P* < 0.001). Larvae experiencing hypoxic exposure + p38 MAPK inhibition were significantly heavier at 2 weeks after HE than 1 week after HE (*P* = 0.008). **(B)** Body length across different time points after HE was significantly longer in controls (*P* = 0.001), and HE + p38 MAPK inhibition (*P* = 0.0002) groups, at 2 weeks after myocardial hypoxic injury, when compared to same groups at 1 week after acute severe hypoxic exposure. Larvae with HE + p38 MAPK inhibition were significantly longer than larvae with acute severe hypoxic exposure alone at 2 weeks after exposure (*P* = 0.005). **(C)** Fulton’s Condition Factor and effect of HE + p38 MAPK inhibition in zebrafish larvae growth. Hypoxic exposure groups with and without p38 MAPK inhibition were significantly higher than controls at 2 weeks after exposure. No significant differences in Condition Factor occurred across different time points after acute severe hypoxic exposure (*P* = 0.78).

Body mass was not significantly different among all three groups at 1 week after HE (*P* > 0.05). However, body mass was significantly higher in all larvae 2 weeks after HE compared to larvae 1 week earlier (*P* = 0.008), showing the normal effects of growth. Body mass in larvae with HE + p38 MAPK inhibition was significantly higher than controls 2 weeks after HE (*P* < 0.001) (Figure 5A). No significant effects in body mass after time occurred in controls and in the HE group.

Total body length across different time points after HE was significantly longer in controls (*P* = 0.001), and HE + p38 MAPK inhibition groups (*P* = 0.0002), at 2 weeks after systemic hypoxic exposure (HE), when compared to same groups at 1 week after HE. Larvae with HE + p38 MAPK inhibition at 2 weeks after HE had a significantly longer body length than larvae with HE only (*P* = 0.005) (Figure 5B).

Regarding the effect of HE and p38 MAPK inhibition in zebrafish larvae growth, HE groups with and without p38 MAPK inhibition had significantly higher Fulton’s Condition Factor than controls at 2 weeks after HE (*P* = 0.005 and *P* = 0.003, respectively). No significant differences occurred across different time points after HE (*P* = 0.78) (Figure 5C).

### 3.3 Time to emerge from start chamber

Latency for emerging from the start (dark) chamber was not significantly different when comparing larval groups at the same time following HE (*P* = 0.99). No significant effect in emergence time from the start chamber occurred as a result of acute severe hypoxic exposure and severe hypoxic exposure + p38 MAPK inhibition when compared from the control group (*P* = 0.96, and *P* = 0.80, respectively).

**FIGURE 6:**
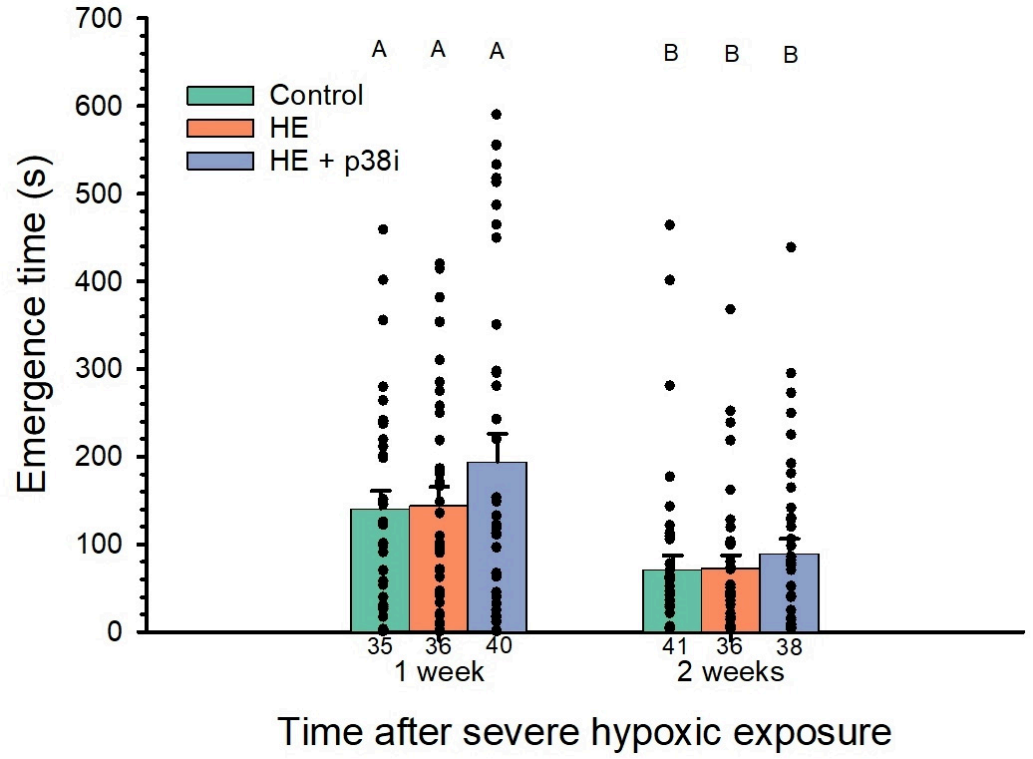
Effects of acute severe hypoxic exposure and p38 MAPK inhibition on time to emerge from the start chamber in zebrafish larvae. N values are at the bottom of each bar. No significant differences occurred among groups within the same week of severe hypoxic exposure (*P* = 0.99). However, there were significant differences between week 1 and week 2 following hypoxic exposure (*P* = 0.03).

However, the time to emerge from the start chamber was significantly shorter in larvae 2 weeks after HE, regardless of experimental group. Time to emerge from the start chamber was significantly shorter in zebrafish larvae at 2 weeks after HE (*P* = 0.03) (Figure 6). Controls at 1 week after HE took 96% longer (*P* = 0.004) to emerge than controls at 2 weeks after HE. Similarly, the HE group took 96% longer to emerge from the start chamber at 1 week after HE, compared to larvae at 2 weeks after HE (P = 0.024). Larvae with HE + p38 MAPK inhibition had a 117% longer emergence time at 1 week after HE than 2 weeks after HE (*P* = 0.03).

### 3.4 Time spent in arena zones

**FIGURE 7:**
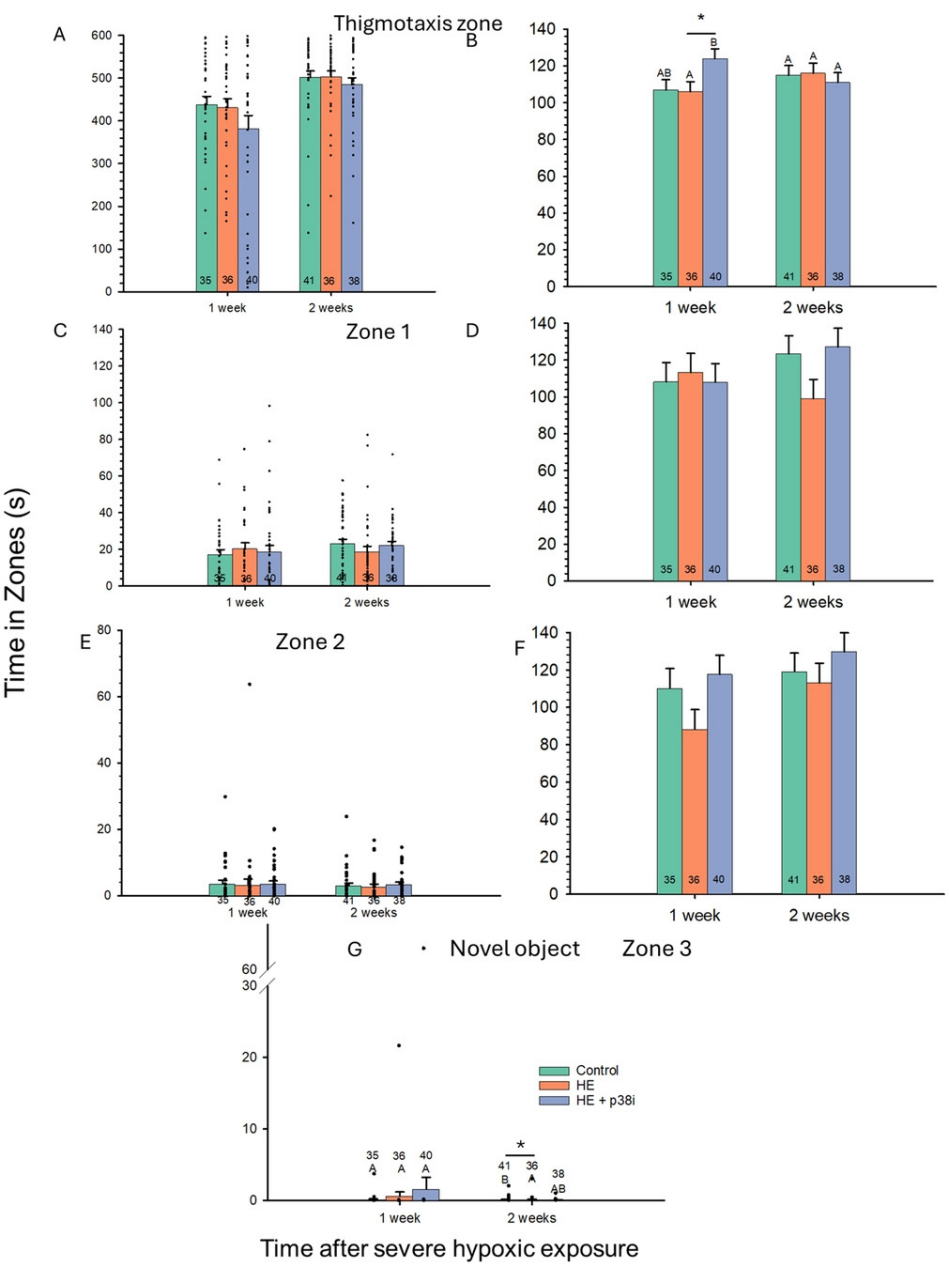
Effects of severe hypoxic exposure and p38 MAPK inhibition on arena zone distribution in zebrafish larvae. N values are at the bottom of each bar. Time of emergence from start chamber was treated as a covariant with the time spent on each zone, except for zone 3 (see text for further information). **(A)** Thigmotaxis zone observed means. **(B)** Thigmotaxis zone estimated marginal means. **(C)** Zone 1 (outer intermedium) observed means. **(D)** Zone 1 estimated marginal means. **(E)** Zone 2 (internal intermedium) observed means. **(F)** Zone 2 estimated marginal means. **(G)** Zone 3 (center zone, novel object zone), observed means. Error bars are plotted as ± S. E. M. *p < 0.05. Different letters indicate significant differences between groups.

Time of emergence from the start chamber was used as a covariant to analyze its effect in the time spent in each of the zones of the arena. p38 MAPK inhibitor had a significant effect on the time spent in the thigmotaxis zone (*P* = 0.02), while the week after HE had no significant effect (*P* = 0.09). Larvae with HE + p38 MAPK inhibition spent significantly more time in the thigmotaxis zone (17% based on estimated marginal means) compared to the HE group with no p38 MAPK inhibition, when considering emergence time as a covariant at 1 week after HE (*P* = 0.04) (Figure 7A). Time spent in the thigmotaxis zone by the control, HE, and HE + p38 MAPK inhibition groups did not differ between 1 and 2 weeks following HE (*P* = 0.25, *P* = 0.2, and *P* = 0.91, respectively). Time to emerge from the start chamber had a significant effect on the time spent in the thigmotaxis zone (*P* = < 0.001).

Time spent in zone 1 was not significantly different between groups (*P* = 0.92) at any point in time, nor across time after HE (*P* = 0.19) (Figures 7C,D). The Treatment Factor was not significantly different in the time spent in zone 2 (Figures 7E,F) and zone 3 (novel object zone) (Figure 7G) (*P* = 0.11, and *P* = 0.94, respectively), nor was the Age Factor after acute severe hypoxic exposure (*P* = 0.41 and *P* = 0.97, respectively). However, the HE group spent significantly less time in the novel object zone, when compared to controls at 2 weeks after HE, when time of emergence from start chamber serves as a covariant (*P* = 0.04). Controls spent significantly longer time in the novel object zone (zone 3) at 2 weeks after HE then controls at 1 week after HE, when emergence time is considered as a covariant (*P* = 0.02). However, the covariant did not have a significant effect (*P* = 0.65), thus, observed means are plotted instead (Figure 7G). Report of the two-way ANOVAs with covariant for the zones 0–3 are included in Table 1.

**TABLE 1:** Results from the general linear model used to analyze the time spent in the different arena zones in zebrafish during acute severe hypoxic exposure assessed with Two-way ANOVA.

Behavioral metric	Intercept	Treatment	Age	Treatment vs. Age	Emergence time
Thigmotaxis*	*F* = 798.60 *P* < 0.001	*F* = 3.90 *P* = 0.02	*F* = 2.89 *P* = 0.09	*F* = 2.98 *P* = 0.05	*F* = 598.57 *P* = < 0.001
Zone 1*	*F* = 116.98 *P* < 0.001	*F* = 0.08 *P* = 0.92	*F* = 1.77 *P* = 0.18	*F* = 1.60 *P* = 0.20	*F* = 21.57 *P* = < 0.001
Zone 2*	*F* = 120.91 *P* < 0.001	*F* = 2.24 *P* = 0.11	*F* = 0.69 *P* = 0.41	*F* = 0.34 *P* = 0.71	*F* = 7.42 *P* = 0.007
Zone 3*	*F* = 336.07 *P* < 0.001	*F* = 0.06 *P* = 0.94	*F* = 0.002 *P* = 0.96	*F* = 1.86 *P* = 0.16	*F* = 0.20 *P* = 0.65
Distance to novel object*	*F* = 10.07 *P* = 0.002	*F* = 0.07 *P* = 0.94	*F* = 0.86 *P* = 0.36	*F* = 0.08 *P* = 0.92	*F* = 8.25 *P* = 0.004

*Indicates rank transformation. Statistical significance was considered with α ≤ 0.05.

### 3.5 Average distance to the novel object—neophilia

**FIGURE 8:**
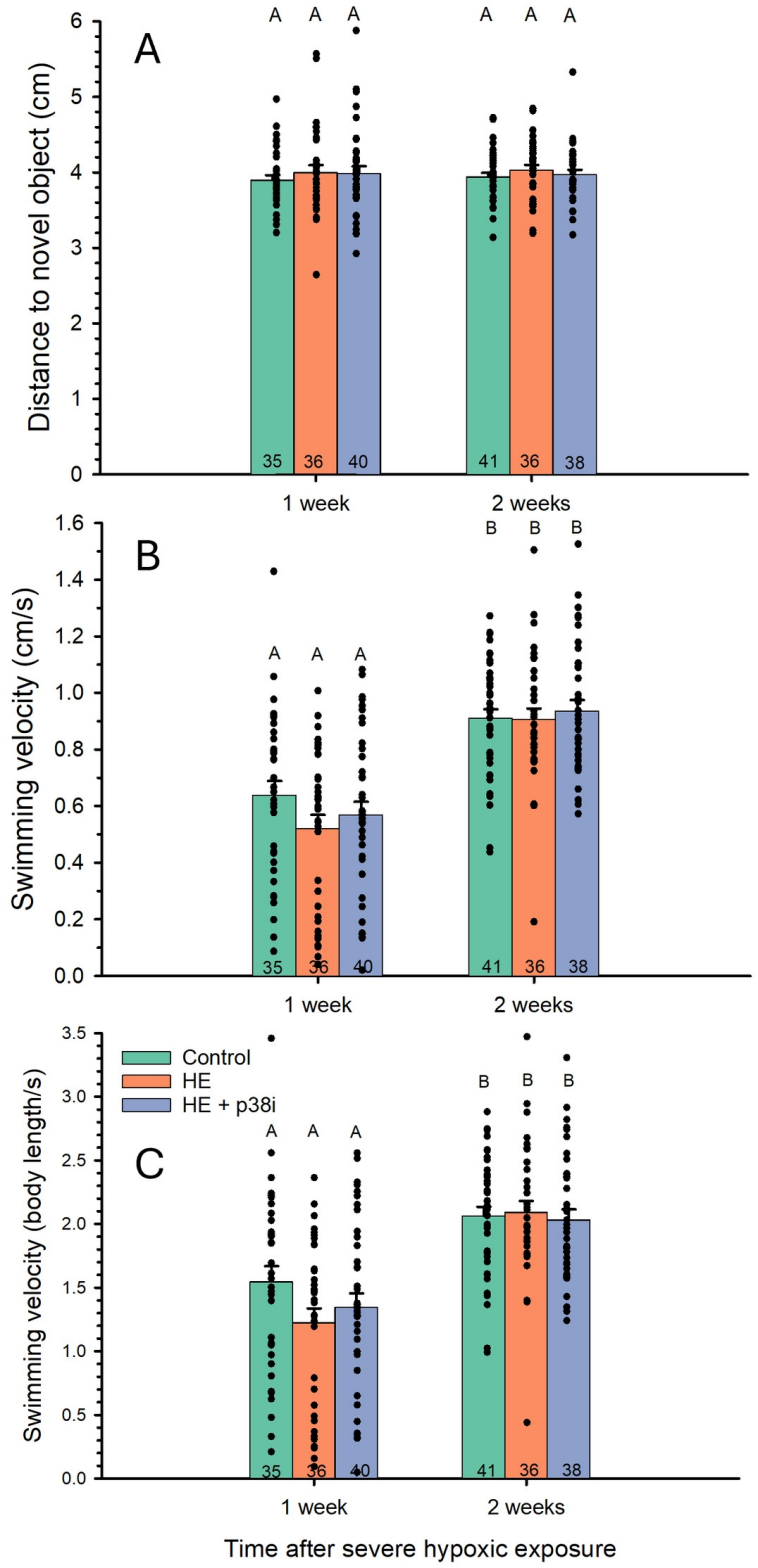
Effects of severe hypoxic exposure and p38 MAPK inhibition on locomotion in larval zebrafish. **(A)** Effects of severe hypoxic exposure and p38 MAPK inhibition on the average distance from the novel object in zebrafish larvae. N values are at the bottom of each bar. No significant differences in distance from novel object occurred between treatments (*P* = 0.936) or with age (*P* = 0.356). **(B)** Effect of severe hypoxic exposure and p38 MAPK inhibition in mean swimming velocity (cm/s) in zebrafish larvae. **(C)** Effect of severe hypoxic exposure and p38 MAPK inhibition in mean swimming velocity (body length/s) in zebrafish larvae. N values are placed at the bottom of the bars. Treatment did not have an effect in mean velocity (*P* = 0.15). However, effects of time after severe hypoxic exposure was significantly different (*P* < 0.001), with larvae swimming more rapidly 2 weeks after severe hypoxic exposure.

No significant differences occurred among groups at the same point in time after HE (P = 0.94), and across weeks after HE (*P* = 0.36) (Figure 8A). Statistics for the two-way ANOVA with covariant for the average distance to the novel object are included in Table 1.

### 3.6 Swimming velocity

The mean swimming velocity of control larvae was 0.64 cm/s at 1 week (14–15 dpf), and 0.91 cm/s at 2 weeks (21–22 dpf). These velocities are equivalent to 1.55 BL/s and 2.07 BL/s, respectively (Table 2). The HE group had a mean velocity of 0.52 cm/s at 1 week after acute severe hypoxic exposure, and 0.91 cm/s 1 week later. The HE + p38 MAPK inhibition group had a mean velocity of 0.57 cm/s at 1 week after HE, and 0.94 cm/s 1 week later. Thus, mean velocity was significantly higher in all larval groups at 2 weeks (*P* < 0.001) (Figure 8B), reflecting the normal developmental growth of the larvae. No significant effect of treatment was observed, with all groups not significantly different when compared at the same time following HE (*P* = 0.15).

**TABLE 2:** Length and velocity indicated as means ± SEM.

Group	Body length (cm)	Velocity (cm/s)	Velocity (BL/s)
Control 8–9 dHE (14–15 dpf)	0.41 ± 0.005	0.64 ± 0.05	1.55 ± 0.12
Control 15–16 dHE (21–22 dpf)	0.44 ± 0.004	0.91 ± 0.03	2.07 ± 0.07
HE 8–9 dHE (14–15 dpf)	0.43 ± 0.005	0.52 ± 0.05	1.23 ± 0.11
HE15–16 dHE (21–22 dpf)	0.43 ± 0.005	0.91 ± 0.04	2.09 ± 0.09
HE + p38i 8–9 dHE (14–15 dpf)	0.42 ± 0.007	0.57 ± 0.05	1.35 ± 0.11
HE + p38i 15–16 dHE (21–22 dpf)	0.46 ± 0.006	0.94 ± 0.04	2.03 ± 0.08

dHE, day after severe hypoxic exposure.

## 4 Discussion

This study provides a comprehensive analysis of chronic changes in survival, growth, and behavioral outcomes in zebrafish larvae resulting from transient, acute severe hypoxic exposure. Moreover, our data show the effect of p38 MAPK inhibition on these variables.

### 4.1 Survival assessment

In a study on larval zebrafish using the same methods for inducing hypoxia, severe hypoxia caused high mortality (Burggren et al., 2024). Surviving larvae exhibited bradycardia and reduced stroke volume, leading to reduced cardiac output. Immunohistochemical examination of heart tissue confirmed increased rates of apoptosis and other markers of cardiac damage. Larval zebrafish exposed to the same degree of acute severe hypoxic exposure in the present study similarly had chronic detrimental effects on survival rate in zebrafish larvae, as expected (Burggren et al., 2024) given the methodological similarities. Burggren et al. (2024) showed survival drops from 90% (5 dpf exposure) to 30% (10 dpf exposure), suggesting reduced regenerative mechanisms at larval stages. In our present study, while control larvae had a higher survival rate compared to larvae acutely exposed to severe hypoxia, larvae with HE and p38 MAPK inhibition had an even lower survival rate than the other groups. These results suggest that p38 MAPK inhibition may exert detrimental effects on survival rate later in development following transient hypoxic exposure. The high mortality in larvae with HE + p38 MAPK inhibition likely involves the complex role of the p38 MAPK pathway in cell survival, inflammation, angiogenesis, and myocardial remodeling (Turner and Blythe, 2019; Yokota and Wang, 2016), the lack of isoform-specific inhibitors, and the adverse effects associated with inhibiting p38 MAPK. For instance, p38 MAPK is involved in several cellular processes such as inflammation and apoptosis (Ren et al., 2005; Romero-Becerra et al., 2020; Wang et al., 1998). Inhibiting these processes may bring unseen adverse side effects later in the ability to achieve cardiac repair, for example (Kompa, 2016; Martin et al., 2015).

The 62% cumulative mortality observed in our control group from 7 to 21 dpf coincides with the well-documented 10–14 dpf developmental bottleneck, during which zebrafish larvae undergo critical physiological transitions (gill development, feeding transition) (Mawed et al., 2019; Rombough, 2002) that frequently result in elevated attrition even in non-experimental cohorts.

### 4.2 Effects on larval growth

As expected, body mass was significantly higher in zebrafish at 2 weeks compared to 1 week after HE, regardless of group treatment, due to developmental growth. Control larvae and HE larvae + p38 MAPK inhibition also showed an increase in total body length over time, but larval length with HE alone was not significantly different for the same treatment at any time. Acute hypoxic exposure *per se* may induce a lower total body length during development, as this group was the only one that did not increase after 1 week. This also may represent an effect of p38 MAPK inhibition, because body mass was also significantly higher in HE larvae with p38 MAPK inhibitor exposure compared to controls. p38 MAPK inhibition may also affect metabolism, because HE larvae + p38 MAPK inhibition weigh significantly more than controls at 2 weeks after HE. However, no significant differences occurred in controls compared to the HE group without p38 MAPK inhibition. p38 MAPK is involved in multiple metabolic processes, such as lipid metabolism and adipogenesis, inflammation and obesity, insulin signaling, and glucose uptake (Leiva et al., 2020; Liu and Cao, 2009). Inhibition of p38 MAPK may disrupt the differentiation of pre-adipocytes into mature fat cells, potentially affecting fat storage and thus body mass. There is no direct evidence of p38 MAPK inhibition treatment causing weight gain after a cardiac ischemic injury in preclinical mammal models. Conversely, p38 MAPK inhibition causes weight loss in mice models (Zhang et al., 2018). p38 MAPK inhibition enhanced the browning of white adipose tissue, considered a metabolic benefit to treat obesity (Zhang et al., 2018).

Fulton’s Condition Factor (K), an indicator of overall body condition in HE larvae, was significantly higher than control groups at 2 weeks after acute severe hypoxic exposure, regardless of pharmacological inhibition (*P* < 0.01). A higher growth rate in HE larvae may result from metabolic imbalance (alterations in ATP homeostasis) (Spelat et al., 2022), because K is significantly higher than control larvae. However, there is no direct evidence of myocardial hypoxic injury causing an increase in organismal growth, as the effects are related to cardiac growth. Cardiac ischemic injury in mammals can cause shifts in energy metabolism of the heart, which may in turn impact the energy balance in the systemic tissues (Martin et al., 2022). Although zebrafish show high cardiac regeneration abilities (Major and Poss, 2007; Poss et al., 2002), effects on larval growth may nonetheless persist after an acute cardiac injury. In mice after myocardial infarction, there are differences across different ages in key molecular regulators (Lindsey et al., 2021). Therefore, growth responses after acute cardiac injury may differ in models according to animal age.

Obesity in mammals is related to inflammation, and the presence of proinflammatory cytokines (Hou and Baldwin, 2012; Milano et al., 2020). In humans, considerable research has been carried out on the relationship of body mass index (BMI), body weight, and myocardial infarction (Ikeda et al., 2011; Joyce et al., 2017; Zeller et al., 2008). Surprisingly, a higher BMI is correlated with a higher survival rate in clinical studies (Ikeda et al., 2011). Nevertheless, the relationship between cardiovascular diseases, mental disorders, and obesity is complex, and more studies in multiple animal models are needed to evaluate the related effects.

Another potential explanation is the presence of edema. Pericardial, yolk sac, or generalized edema is a well-documented pathological response to hypoxic stress and pharmacological perturbations in developing zebrafish (Chen, 2013). Fluid accumulation increases body mass without reflecting true somatic growth. The elevated Fulton’s Condition Factor in HE groups (Figure 5C) supports disproportionate mass relative to length, consistent with edema. We did not systematically assess pericardial or yolk sac edema in fixed larvae, representing a limitation. If increased mass primarily reflects fluid accumulation, this would indicate pathological rather than beneficial effects of p38 inhibition. Edema could contribute to higher mortality through cardiac compromise or impaired systemic circulation (Boyle et al., 2007).

### 4.3 Chronic behavioral effects after acute severe hypoxic exposure

Time of emergence from the start chamber is associated with risk-taking, used as a proxy for boldness in fishes (Toms et al., 2010). Emergence time is affected by development alone, because zebrafish larvae took less time to emerge at 2 weeks compared to after just 1 week, with no difference among the three experimental groups. Zebrafish larvae naturally grow bolder as developmental time increases (Figure 6). Neither acute severe hypoxic exposure nor p38 MAPK inhibition in zebrafish larvae chronically influenced time to emerge from the start chamber. Changes within treatments occurred after time, with a shorter emergence time in older larvae. Collectively, these data indicate the natural increase in boldness in zebrafish (Alfonso et al., 2020).

Boldness has been associated with the exploratory-boldness behavioral syndrome in zebrafish (Wisenden et al., 2011), associated with increased exploration (Norton and Bally-Cuif, 2012). Boldness is also considered to be risk-taking behavior, and bolder fish have increased neophilia (White et al., 2013). In our study, control larvae became significantly bolder during normal development from 14 to 15 dpf to 21–22 dpf, spending more time in the novel object zone as they grew older. Thus, neophilia increased with developmental rate in zebrafish. Regardless of the increased time spent in the novel object zone, the average distance to the novel object did not significantly change among groups.

Notably, HE larvae without p38 MAPK inhibition spent significantly less time in the novel object zone—that is, were less inquisitive—than controls, at 2 weeks after exposure, when time of emergence from the start chamber was made a covariant. Few studies address changes in boldness behavior after severe hypoxic exposure, often associated with acute cardiac syndromes (ACS) such as myocardial infarction, in any animal model or humans. Decision-making and risk-taking are directly influenced by boldness behavior in humans and other animals (Sloan Wilson et al., 1994). Further studies are required to understand the implications of tissue injury, including cardiac injury and other cardiac pathologies, on boldness behavior.

The low absolute time in zone 3 across all groups may reflect age-appropriate caution in 14–21 dpf larvae, where brief investigative approaches followed by retreat to safer peripheral zones represent adaptive behavior. Future studies should incorporate: (1) positive controls using validated neophilia-modulating compounds to establish expected effect size ranges, (2) multiple behavioral paradigms to provide convergent evidence (e.g., light-dark preference, shelter-seeking), and (3) ecological validation experiments examining whether observed behavioral differences predict differential outcomes under predation pressure or resource competition. The absolute magnitude of these changes—approximately 1–2 s difference in zone occupancy during a 600-s trial—raises valid questions about ecological and functional relevance. It remains unclear whether these alterations would translate to measurable differences in predator avoidance efficiency, foraging success, social integration, or long-term developmental outcomes. To our knowledge, no established benchmarks exist in the larval zebrafish literature defining the minimum behavioral change magnitude required to impact fitness-related outcomes.

Reduction of physical activity is a common side effect of myocardial infarction in humans. Fatigue, and shortness of breath may induce sedentarism and decrease boldness behavior to avoid complications. Zebrafish larvae with heart failure showed a decrease in distance traveled (Kim et al., 2022). Unfortunately, there are few clinical studies explicitly addressing the link of cardiac injury to boldness behavior.

Swimming velocity was significantly higher in the older larval zebrafish in our study. This was expected because in an ecological context, a higher velocity confers greater ability to avoid predation, capture prey, and exploration of the environment. Locomotion variables are also useful for assessing anxiety-like behavior in experimental animal models such as fish, from a clinical perspective (Hagen et al., 2024). Increased velocity but also immobility are proxies for anxiety, creating complex interpretations because locomotion can be challenged to measure anxiety-like behavior (Johnson et al., 2023). However, in the current study, HE + p38 MAPK inhibition did not alter mean swimming velocity (Figures 8B,C). The absence of velocity or emergence time alterations suggests that hypoxic exposure and p38 inhibition did not produce global locomotor impairment, but rather selective changes in exploratory and anxiety-related behaviors. Whether these selective behavioral alterations carry ecological or clinical significance—such as reduced survival under predation pressure or altered developmental trajectories into adulthood—requires future investigation correlating behavioral phenotypes with fitness outcomes. Our findings suggest alterations in exploratory and anxiety-like behaviors, but assessment using a single behavioral paradigm (novel object approach with thigmotaxis quantification) limits definitive conclusions. Convergent evidence from additional validated assays—such as light-dark preference, acoustic startle response and habituation, or social interaction tests—would strengthen characterization of the behavioral phenotype and determine whether effects generalize across multiple anxiety-related behavioral domains.

Importantly, mechanisms regulating anxiety and boldness are evolutionarily conserved, being very similar from fish to mammals (Colwill and Creton, 2011; Fontana et al., 2021; Richendrfer et al., 2012). Anxiety-like behavior occurs very early in zebrafish development, evident from a strong preference for larvae to stay in the thigmotaxis zone—i.e., the edge of the arenas (Schnörr et al., 2012) even with a few days after hatching (Richendrfer et al., 2012).

Our study indicates that anxiety-like behavior is not chronically affected by acute severe hypoxic exposure in zebrafish, because HE larvae did not differ significantly from the controls at either of the assessed time points. However, larvae with HE + p38 MAPK inhibition spent significantly more time in the thigmotaxis zone than the larvae with hypoxic exposure alone, when emergence time is used as a covariant. This could represent an anxiogenic effect for p38 MAPK inhibition, but these mechanisms have not yet been studied. Interestingly, anxiety produced by sleep deprivation in mice was reduced by inhibiting p38 activation and reducing the inflammatory response (Li et al., 2023). p38 MAPK has multiple functions in the central nervous system, involved mainly in neuroinflammation (Corrêa and Eales, 2012), nociception (Asih et al., 2020; Mai et al., 2020), synaptic plasticity and cognitive function (Falcicchia et al., 2020). Also, p38 MAPK is implicated in decision-making in different animal models such as mice (Asih et al., 2020). Deletion of p38α in neurons increases anxiety in mice (Asih et al., 2020; Stefanoska et al., 2018). This mechanism is related to the inhibition of c-Jun N-terminal kinase by p38α in mice neurons (Stefanoska et al., 2018).

There is clear evidence of comorbid anxiety after myocardial infarction in humans (Geiser et al., 2017; Liblik et al., 2022; Macit et al., 2009; Murphy et al., 2015). Nonetheless, others have proposed anxiety as an adaptive behavior, inducing higher medical compliance in patients with anxiety post-MI (Benninghoven et al., 2005; Parker et al., 2011). However, anxiety is considered a stress response. The stress theory explains the relationship between psychological factors and cardiovascular diseases, in which the stress response plays a critical role (Chauvet-Gelinier and Bonin, 2017). Oxidative stress is caused by the overactivation of the SNS, and the activation of the hypothalamic-pituitary-adrenal (HPA) axis (Repova et al., 2022), homologous to the hypothalamic-pituitary-interrenal axis in zebrafish (Wei et al., 2020).

Noteworthy is that animal models are not completely optimal for mimicking cardiovascular diseases in humans, due to the heterogenicity of pathological factors in the human population (Papamichail et al., 2023). Certainly, the use of zebrafish has its limitations when translating findings to the clinical field (Phillips and Westerfield, 2014). An example is the great differences in regenerative potential in some organs such as the heart (Lien et al., 2012; Poss et al., 2002; Stewart et al., 2022; Zuppo and Tsang, 2020) and the brain (Kishimoto et al., 2012; Kizil et al., 2012). Furthermore, zebrafish cardiac regeneration is age-dependent (Reuter et al., 2022).

Another complication is the difference in drug absorption in zebrafish and humans, because the primary route of drug administration in zebrafish is by aquatic exposure, which differs from normal drug administration routes in mammals (Tal et al., 2020). A possible critique of the methodology is that the drug was administered globally rather than being targeted to a specific tissue. This could induce systemic effects that may confound the interpretation of the results. Moreover, systemic hypoxia affects multiple organ systems, not exclusively the heart, contributing to neurobehavioral outcomes.

Future experiments should consider localized delivery methods, such as cell-specific targeting techniques (Zhao et al., 2020). This approach would help to isolate its effects and provide a better understanding of its specific mechanisms. Furthermore, some techniques based on machine learning contribute to a more holistic in depth analysis of anxiety-like behavior in fish (Muralidharan et al., 2024; Srivastava et al., 2025). Those techniques are proposed to be considered in future studies to robust the characterization of anxiety-like behavior.

Another potential limitation of the present study is the absence of direct cardiac functional measurements—such as heart rate, stroke volume, and cardiac output—within the specific cohorts subjected to behavioral analysis. We have reasonably inferred myocardial damage based on standardized protocols previously validated to induce cardiac arrest and apoptotic injury in zebrafish larvae (Burggren et al., 2024). However, the experimental design of our current study precludes the ability to correlate individual injury severity or specific recovery trajectories with observed behavioral phenotypes. Acute severe systemic hypoxia causes oxidative damage across multiple tissues, including the brain, so the behavioral changes reported here may result from a combination of cardiac-mediated systemic effects and direct neurotoxic insults. To better isolate these mechanisms, future research should integrate real-time echocardiographic or hemodynamic assessments with behavioral assays to establish whether the magnitude of neurobehavioral alteration scales directly with the severity of cardiac dysfunction. Finally, an important limitation of our experimental design is that behavioral testing occurred at two developmental time points (14–15 dpf and 21–22 dpf) when zebrafish larvae undergo substantial physiological and behavioral maturation. This is evident in our control data showing developmental increases in emergence speed (reduction in latency), swimming velocity, and novel object exploration independent of treatment effects. Because developmental changes are confounded with time-after-exposure in our design, it is challenging to distinguish delayed effects of acute hypoxic injury from normal developmental trajectories that were modified by early-life injury. However, the differential effects of hypoxic exposure (with or without p38 inhibition) at the same developmental time points suggest treatment-specific alterations superimposed on normal developmental progression. For example, at 21–22 dpf, control larvae increased novel object zone occupancy compared to 14–15 dpf (normal developmental boldness increase), whereas HE larvae did not show this developmental progression—suggesting that early hypoxic exposure impairs the normal trajectory of exploratory behavior maturation. Future studies using adult zebrafish, where behavioral and physiological baselines are stable, would eliminate developmental confounding and allow clearer interpretation of chronic effects following acute hypoxic injury. Adult studies would also permit assessment of whether early-life hypoxic exposure produces lasting behavioral alterations into adulthood, or whether effects are limited to periods of active growth and development.

## 5 Conclusion

Acute severe hypoxic exposure significantly affected boldness behavior in zebrafish in our study, with the HE group spending 21% less time than control groups in the novel object zone. p38 MAPK inhibition significantly increased thigmotaxis, suggesting enhanced anxiety-like behavior in zebrafish larvae, because HE larvae with p38 MAPK inhibition spent 17% more time in the thigmotaxis zone than larvae exposed to acute severe hypoxia with cardiac injury but no p38 MAPK inhibitor exposure. Time to emerge from the start chamber and velocity were only affected by development, as there was no significant effect of acute severe hypoxic exposure or p38 MAPK inhibition.

This study makes several important contributions: (1) it is the first to examine chronic neurobehavioral consequences of acute early-life hypoxic injury in zebrafish larvae, establishing proof-of-concept for this translational model; (2) it identifies p38 MAPK inhibition as having complex, potentially opposing effects on growth and behavior, informing therapeutic development efforts; and (3) it highlights the need for integrated assessment of survival, growth, and behavioral endpoints in developmental toxicology and pharmacology studies. The behavioral effects are modest, do not affect all aspects of behavior and their functional significance requires further validation. The persistent, selective nature of some of the alterations suggests that acute severe hypoxia during critical developmental windows produces lasting changes in neural circuits governing exploratory behavior and anxiety-related responses.

These findings highlight the need for a holistic approach that establishes temporal assessment windows that capture both acute regenerative phases and long-term functional outcomes needed to identify critical intervention points and optimize therapeutic timing. By bridging the gap between cellular mechanisms and organismal behavioral phenotypes, these dual-assessment approaches can inform precision medicine strategies that address both the physiological damage and behavioral consequences of acute severe tissue hypoxia accompanying cardiac injury.

## Data Availability

The raw data supporting the conclusions of this article will be made available by the authors, without undue reservation.
